# P-385. A Change of Heart: Limitations of National Healthcare Safety Network (NHSN) and Society of Thoracic Surgeons (STS) Surveillance Systems in Identifying SSI After Heart Transplant Surgery

**DOI:** 10.1093/ofid/ofae631.586

**Published:** 2025-01-29

**Authors:** Jessica Seidelman, Becky A Smith, Sana Arif, Sarah Lewis, Erin Gettler, Polly W Padgette, Brittain A Wood, Melissa Williams, Peter Fleming, Jacob Schroder, Carmelo Milano, Rachel Miller, Barbara D Alexander, Manuela Carugati

**Affiliations:** Duke University School of Medicine, Durham, North Carolina; Duke University, durham, North Carolina; Duke University, durham, North Carolina; Duke University, durham, North Carolina; Duke University Medical Center, Durham, NC; Duke Infection Control Outreach Network (DICON), KNIGHTDALE, North Carolina; Duke Infection Control Outreach Network (DICON), KNIGHTDALE, North Carolina; Duke University, durham, North Carolina; Duke University, durham, North Carolina; Duke University, durham, North Carolina; Duke University Hospital, Durham, NC; Duke University, durham, North Carolina; Duke University School of Medicine, Durham, North Carolina; Duke University, durham, North Carolina

## Abstract

**Background:**

Surgical site infections (SSI) complicate 4.8-12.4% of orthotopic heart transplants (OHT) and result in increased length of hospital stay, mortality, and health care costs. As such, the need for accurate SSI surveillance systems for OHT is critical. This study aimed to evaluate the diagnostic accuracy of the National Healthcare Safety Network (NHSN) and Society of Thoracic Surgeons (STS) surveillance systems for SSI against the gold standard of a detailed manual adjudication process performed by Transplant ID physicians (TXID).

**Methods:**

We retrospectively reviewed single-organ OHT performed at an academic medical center between 1/1/19 and 12/31/20 among adult patients (≥18 years). Each OHT was adjudicated for SSI by NHSN, STS, and TXID SSI definitions. The TXID definition was the same as the NHSN classification. The surveillance window was 90 days for NHSN and TXID. STS adopted a 90-day surveillance window for deep sternal wound infections from 7/1/20 onward.

**Results:**

TXID identified 17 (10.5%) SSI among 162 OHT surgeries during the study period. NHSN identified only 7 (4.3%) SSI, including 3 false positive SSI, which were not considered SSI by TXID. STS identified only 4 (2.5%) SSI during the study period and no false positive SSI(Fig 1). The sensitivity, specificity, positive-predicted-value, and negative-predicted-value for NHSN were 23.5%, 97.9%, 57.1%, and 91.6% and for STS 23.5%, 100%, 100%, and 91.8%, respectively. The suboptimal sensitivity of NHSN was mostly explained by intervening surgeries (such as chest closures or washouts) that prevented a subsequent SSI from being attributed to the OHT, and by STS’s prior 30-day surveillance window.

**Conclusion:**

Accuracy of SSI surveillance is essential for SSI prevention. Current SSI surveillance systems have low sensitivity in detecting OHT SSI, which is likely due to the complex nature of the hospital courses following OHT surgeries. While a manual adjudication performed by Transplant ID physicians is time- and resource-intensive, it likely provides a more accurate depiction of SSI rates compared to automated surveillance systems. Future studies are needed to determine how NHSN and STS surveillance systems can be adjusted to more accurately identify OHT SSI.

**Disclosures:**

**Jessica Seidelman, MD, MPH**, 3M: Expert Testimony **Becky A. Smith, MD**, UpToDate: royalties **Jacob Schroder, MD**, Abbott: Advisor/Consultant|Abiomed: Advisor/Consultant **Barbara D. Alexander, MD**, Basilea: Advisor/Consultant|F2G: Advisor/Consultant|F2G: Grant/Research Support|HealthTrackRx: Advisor/Consultant|HealthTrackRx: Board Member|Scynexis: Grant/Research Support|TFF Pharmaceuticals: Advisor/Consultant

